# A qualitative content analysis of the experience of living with hypospadias: varying impact on identity and interpersonal relationships

**DOI:** 10.3389/fped.2024.1459561

**Published:** 2024-08-29

**Authors:** Lottie Phillips, Nicklas Dennermalm, Lisa Örtqvist, Hedvig Engberg, Gundela Holmdahl, Magdalena Fossum, Anders Möller, Agneta Nordenskjöld

**Affiliations:** ^1^ Department of Women’s and Children’s Health and Centre of Molecular Medicine, Karolinska Institutet, Stockholm, Sweden; ^2^ Department of Social Work, Stockholm University, Stockholm, Sweden; ^3^ Department of Paediatric Surgery, Astrid Lindgren Children’s Hospital, Karolinska University Hospital, Stockholm, Sweden; ^4^ Department of Gynaecology and Reproductive Medicine, Karolinska University Hospital, Stockholm, Sweden; ^5^ Department of Paediatric Surgery, Rigshospitalet and Faculty of Health and Medical Sciences, Copenhagen University, Copenhagen, Denmark; ^6^ School of Public Health and Community Medicine, Institute of Medicine, University of Gothenburg, Gothenburg, Sweden

**Keywords:** hypospadias, qualitative content analysis, in-depth interview, patient perspective, disorders of sex development

## Abstract

**Objectives:**

There is a lack of in-depth studies on men's personal experiences of having hypospadias across different aspects of their lives. We therefore aimed to explore the experience of having hypospadias in relation to identity and interpersonal relationships.

**Subjects and methods:**

Using purposive sampling, we included 17 adult men aged 20–49 with variation in hypospadias phenotype. The informants further represented variation in sexuality, relationship status, parental status, and familial cultural context. In-depth interviews were conducted with each informant and the data was analysed using qualitative content analysis.

**Results:**

We identified four categories. Firstly, *The internal* e*xperience of hypospadias* in relation to being different, being impacted, and being masculine. The remaining three categories related to interpersonal spaces: *Intimate spaces*, comprising personal relationship with sex, having sex, and being in a relationship; *Familial spaces*, comprising being a son, and becoming a father; and *Public spaces*, comprising being hidden, being naked, and peeing. We identified the latent theme *varying impact and coping*, highlighting differences in experiences relating to both the internal and interpersonal.

**Discussion:**

Issues related to hypospadias included struggles with identity and confidence, as well as recurring patterns of social and sexual avoidance. While informants generally related to certain shared experiences, there is large variation in how much hypospadias impacts life, ranging from hardly at all to extensively. This could also fluctuate over time, with puberty and adolescence being an especially sensitive period. Functional and aesthetic outcomes are potentially important for well-being, especially in the case of more severe complications, while personal and interpersonal circumstances play a role in coping and the overall experience of the individual.

**Conclusion:**

Healthcare, research, and other channels such as patient groups may be able to offer support to those who need it to help more boys and men with hypospadias live unhindered lives.

## 1 Introduction

Hypospadias is present in around 1 in 125 boys, resulting from a disruption in the development of the penis *in utero* (1). The foreskin is typically cleaved and hooded, and the urethra does not extend to the tip of the glans. Most cases are distal, with a urethral opening on the underside of the glans penis or the distal penile shaft, while in around 10–15% of cases the opening is by the penile base, the scrotum, or the perineum (2). Some boys also have a ventral penile curvature, more commonly in proximal hypospadias. Penile length is on average decreased, sometimes significantly (3).

The underlying aetiology is complex and not yet fully understood, with some cases having more direct genetic causes and others resulting from environmental factors during pregnancy or a combination of different risk factors (4). Testosterone and other androgens play a key role, and some boys have other conditions related to low testosterone function such as undescended testes (5). Hypospadias can be heritable, depending on the cause, and there is a recurrence risk in offspring and brothers (6).

Physical outcomes in men born with hypospadias are generally good, although more long-term outcomes are less studied overall (7). Most cases are treated surgically in early childhood, aiming to extend the urethra to the tip of the penis and correct penile curvature. Complications include persistent curvature and urethral strictures and fistulas leading to voiding dysfunction (3, 8, 9). However, techniques have developed in recent decades and distal hypospadias is generally associated with a low risk of complications, with a significantly higher risk in proximal hypospadias (10, 11). Complications can occur or become apparent well into adulthood (11). Larger-scale epidemiological studies have further indicated somewhat impaired fertility, especially for proximal hypospadias, and more androgen-related comorbidity (12–14).

Swedish studies have found that men with hypospadias have largely equivalent social outcomes, such as education and income, on a population level, and are as likely to get married as their peers (15, 16). However, fear or avoidance of intimacy and relationships has been reported, as well as avoidance of for instance public showers (17). Men with proximal hypospadias in particular have also been shown to have an on average decreased satisfaction with their penile appearance and reports of sexual dysfunction have included issues with ejaculation, erection, and reduced sensation (16, 18).

We aimed to further explore the personal experience of living with hypospadias using qualitative methods and have previously published our results relating to hypospadias care (19). Here we present our results relating to identity and interpersonal relationships.

## 2 Materials and methods

We selected a qualitative study design in order to further explore the personal experience of living with hypospadias. In-depth interviews were chosen given the sensitive and personal subject matter. The qualitative content analysis of our interview data provided extensive results which we chose to present in two separate articles. Further detailed methodology, as well as results from our study that related to the experience of hypospadias care, have been previously published (19).

Ethical permission was granted by the Swedish Ethical Review Authority (reference No. 2008/1671-31/3 and 2018/1875-32). All participants signed written consent forms and were able to withdraw their consent during the study.

### 2.1 Study setting and informants

During the study period in our Scandinavian setting, gender-specific public showers and changing rooms have been commonplace throughout physical education at school, as well as organised recreational sporting and outdoor activities. Male circumcision is rare in most of Sweden with an estimated total number (across all ages) of around 3,000 annually (total population of circa 10 million), although this number has likely changed over time with increased immigration (20). Details of hypospadias care in Sweden have been previously described (19). In brief, most boys with hypospadias in Sweden have been treated surgically within our universal free healthcare system, typically between the ages of two and six years old depending on the time period.

In total, 17 adult informants were recruited aged 20–49, primarily by contacting former patients from Stockholm, Sweden as well as other methods including personal knowledge and self-referral (19). Recruitment of informants stopped when the two interviewers agreed upon data saturation, defined as no significant new information, relating to the study aim, in variation or depth. Amongst our informants, phenotypes varied across a large spectrum including glanular hypospadias, penoscrotal hypospadias, and small penis (less than 2.5 SD from the mean), with multiple informants across each part of the spectrum. All informants identified as men and stated sexualities included hetero-, homo-, and bi- or pansexual. Current relationship status varied from no prior intimate relationships to married or separated. Around half of the informants had biological children at the time of interview. There were varying levels of education and types of occupation (i.e., student, unemployed, or different types of employment). Finally, there was variation in sociodemographic background including familial culture and religion as well as growing up in larger cities or smaller communities.

### 2.2 Interviews and data analysis

In-depth interviews were conducted with each informant between May 2019 and August 2021 and recorded in full, lasting around 40 min on average range: (14–68 min). The interviewer was either the first or second author. All interviews followed an interview guide starting with an open question about the experience of living with hypospadias, and further covering the topics of childhood, healthcare, identity, relationships, and fatherhood (19). Informants were allowed to say as much as they wished about each topic and were asked if they had anything more to add at the end.

**Figure 1:**
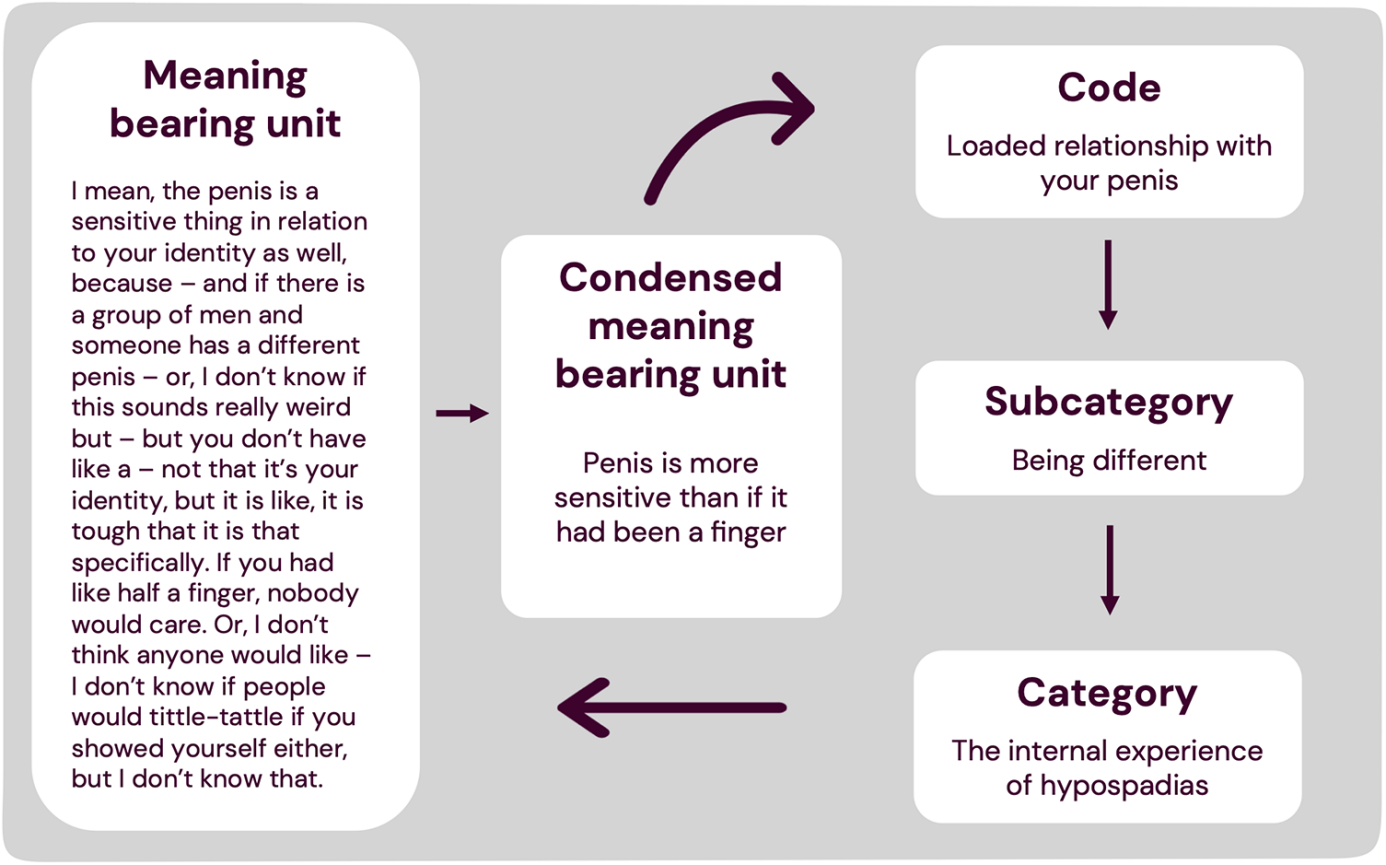
Example of data analysis. Example from the results of how a section of text was condensed into a condensed meaning bearing unit and abstracted into a code, subcategory and category. The bottom arrow pointing left illustrates that the process was iterative and repeated until the fullest possible comprehension of the data was achieved.

All recorded interviews were transcribed verbatim and analysed using qualitative content analysis (21). Meaning bearing units in each interview were identified and abstracted to form condensed meaning bearing units, codes, subcategories, and categories (Figure 1), using Nvivo (Release 1.7) to assist data handling. The analysis was primarily inductive, i.e., arising from the data, but with repeated interpretation from the collective diverse expertise and experience of the research group (19). The results were also assessed for any latent overarching themes across categories.

## 3 Results

**Figure 2:**
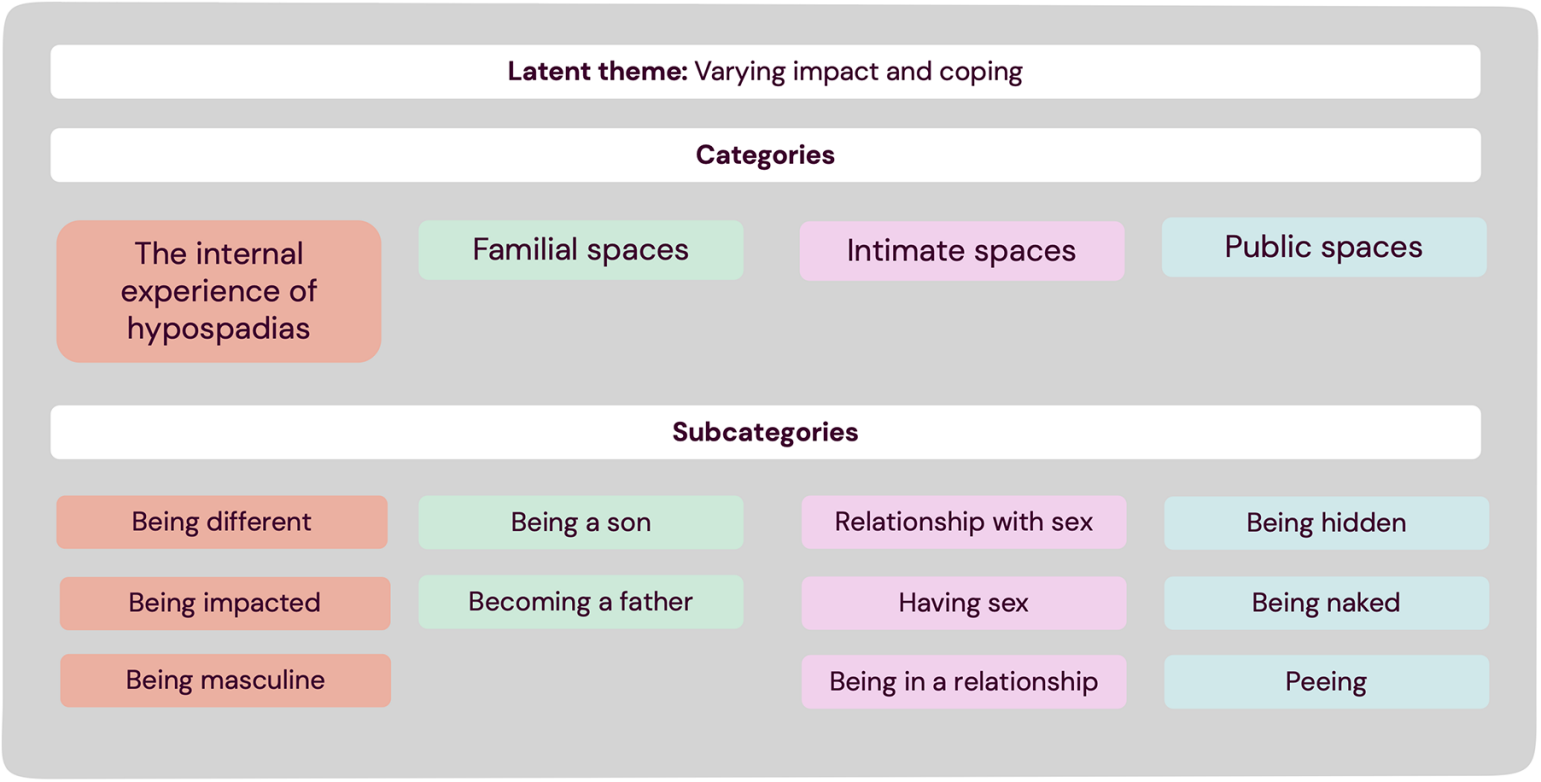
Results of qualitative content analysis.

We identified four categories in our analysis: “*The internal experience of hypospadias”*, as well as experiencing hypospadias in relation to three external spaces: “*Familial spaces*”, “*Intimate spaces*”, and “*Public spaces*”. We also identified a latent theme of *varying impact and coping* (Figure 2).

### 3.1 The internal experience of having hypospadias

This category comprises aspects relating to personal identity, including both being and feeling different, being impacted overall by hypospadias and being masculine.

#### 3.1.1 Being different

Our informants described a process of reaching self-awareness, usually when approaching the pre-pubertal period, when they started to consider their identity and bodies, and the ways in which they differed from their peers.

Many physical differences were described, mostly related to the penis but also for instance being shorter in height or having a different pubertal development. When describing how their penis was different, not having a foreskin, or having an unusual foreskin, and smaller penile size were recurringly described as more obvious differences. Others included the shape and curvature of the penis and glans, the size, angle, or shape of the urethral opening, scarring, and the overall aesthetic appearance. Some informants described their penis as not being “*beautiful*” or looking “*malformed*”. Size in particular was a focal point, though informants varyingly described their own perceived size across a spectrum from small to large.

Apart from more direct physical differences, informants related in some way to a sense of being different, varying across a spectrum from fearing being, rather than feeling, different, to having a strong sense of otherness: “*All forms of feeling alienated, alienation, was connected to malformation. To a feeling deep inside that ‘I’m born that way'” (IN12)*. Being different was generally viewed as difficult, or even directly negative or bad, and could induce feelings of stigma or shame. One informant suggested preventing these identity issues by instilling the concept of normal from an early age: “*It was more uncomfortable for me, because I had (…) no confirmation that it was normal or functioning or anything. Well, the point is that you sort of settle in with that this is normal, and then you're ok with it, but if you don't know when you're a child, it's a problem*” *(IN15)*.

When talking about being different, it was described as particularly difficult and sensitive that the penis was the affected body part. While it was discussed that a complex relationship to your penis at some point in life may be ubiquitous, hypospadias added another layer of complexity. When asked how they felt about their penis today, some informants were more neutral or content. For instance, being circumcised could be viewed as positive. Others struggled with negative emotions, feeling envy or regret about the way they look: “*I think it has been very difficult, anyway, being different, and it has- it has impacted me all my life, in a negative way. (…) It is like you are expected to look a certain way, and if you are different from that…” (IN14).*

#### 3.1.2 Being impacted

Some informants felt that hypospadias had not had any significant impact on their well-being or the way they lived. However, others described hypospadias as being at the heart of insecurities and decreased well-being in their lives: “*I had to learn to live with a different looking, deformed, circumcised, scarred, slightly bent, and often painful sexual organ when I peed and later on when I had wet dreams or masturbated*” *(IN15, added in written correspondence and included in analysis with consent)*.

When informants attempted to hypothesize why hypospadias had a big or small impact on their lives, the reasons they mentioned included functional and aesthetic outcomes, but also their own personality or psychology: “*But then, I am an optimist and a positive person, so I think that helps too. I don't know what it's like, on average. How others with [hypospadias] generally feel” (IN10)*. Another factor was how you are treated by family, friends, or partners: “*I have been very lucky. I have had really great friends in my life, I have- great girls and no, hypospadias has not stopped me from living a good life” (IN03)*.

Puberty was highlighted as the time when hypospadias can have a particularly large impact. Learning to accept hypospadias with age was described as part of letting insecurities go as you grow, or as the result of reinforcing positive experiences. Some informants said that they rarely thought about hypospadias now as adults. However, the impact of hypospadias could also worsen in adulthood, with greater understanding of physical issues. Acceptance could be hard to reach: “*It's like being born in an up-hill battle. So that is what I have found hard to accept. I have always wished to be someone else” (IN12)*.

Some informants discussed whether they prescribed hypospadias a larger role than it had, describing it as a sort of emotional scapegoat: “*But the question is what is what, connected to the things that have been hard. But I can say that hypospadias hasn't helped. But I have probably blamed quite a lot on it and have for a very long time had it as my big complex, when I have daydreamed myself away from it, in my thoughts- daydreamed myself away from having any forms of deformities relating to my penis” (IN12)*. Others felt that hypospadias had a smaller impact than other issues: “*If you were to list reasons, then hypospadias is part of it. (…) It is not something that can like- it doesn't increase your confidence- like you can't increase your confidence by having hypospadias, but at the same time, it doesn't take away from anything. (…) I have a lot of other problems. Hypospadias is like the smallest problem I have or have had” (IN07)*.

#### 3.1.3 Being masculine

Some informants felt that hypospadias had no relation to how they felt as a man, while others felt insecure or inadequate. For those who felt insecure, differences in physical development discussed above could be perceived as signs of being under-masculinised. Being overtly masculine was not always viewed as important or desirable, and feelings around masculinity and hypospadias could fluctuate with time and circumstance. For instance, some felt that feelings of inadequacy came to the surface around women, while others felt more insecure in comparison to other men: “*I feel that I've struggled to assert myself and feel fully like a man. (…) I have had [some] kids so I am fully normal in a lot of ways, but it is deep rooted in my core that I struggle to feel completely at ease and to approach other men as an equal.” (IN12)*.

### 3.2 Familial spaces

This category comprises experiences related to hypospadias in familial spaces, both with your own parents and related to becoming a father.

#### 3.2.1 Being a son

How parents were perceived to have related to their son's hypospadias varied from very open and supportive, to closed off, with informants feeling that the subject was “*taboo*”. It was suggested that some parents would have benefited from more support in learning how to approach it. However, some informants felt that they themselves did not want to talk about their hypospadias, feeling it was too intimate and personal, especially growing up. In more extreme cases, parent's behaviour or comments could worsen a sense of shame, while others associated an actively supportive and normalising parent with improved well-being and confidence: “*I don't think that it has been that traumatic for me [having hypospadias]. But (…) I should probably say that I have been lucky too, in that I had my mother too, who was so good and understanding and, yeah, sensitive in the right way. She supported me in, whatever came up surrounding that, very well” (IN17)*.

The mother was highlighted as the primary figure who handled communication, support, and practical aspects. While having a father or other male family members with hypospadias could be viewed as comforting or normalising, and it was discussed that fathers in theory could relate more directly to their son's struggles, it was generally still the mother who played the key role.

Parents were also a source of information, sometimes willingly answering questions and in other cases seemingly avoiding or withholding things. Having parents who worked in healthcare themselves could mean access to more information: “*[They] knew some stuff and could read up as well and then process that information pretty well. I can imagine that in other-yeah but like in a family where it is not as easy to find information and maybe understand what it means and so on, then maybe there would have been more unanswered questions” (IN08)*.

#### 3.2.2 Becoming a father

Some of our informants had not yet even thought about kids, whereas others had fathered multiple children. None had had trouble conceiving. Fertility was varyingly not thought about at all, or an area of concern: “*I think I thought that I was unsure at first, if I even could have kids. I don't know if- you don't know how you are affected in different ways. If you then like feel unmasculine, then you wonder ‘what is the quality of my sperm?’ I have no idea” (IN12)*. Some informants had concluded that they should be fine given they had no functional issues, or that fertility is generally unrelated to hypospadias.

More prominent was a concern about heritability, although the idea that hypospadias could been inherited might not have even occurred before having a son who was affected. While the fear of having a son with hypospadias was so great for some informants that it was deterring, others felt it had no impact on paternal interest, although they hoped that their children would not be affected. After having children, informants described an increased awareness of how they peed: “*I have thought about it, now that I have started looking into it again, if it is heritable. But I guess that is what I will get answered at the doctor's that I got a referral to see. And if there is something that I should keep an eye out for with my kids, but both my children seem to pee normally” (IN05)*.

### 3.3 Intimate spaces

This category comprises experiences related to hypospadias in intimate spaces, including our informants own personal relationship with sexuality and intimacy, as well as having sex with partners and being in a relationship.

#### 3.3.1 Personal relationship with sex

The sexual impact most clearly described in relation to hypospadias was apprehension and avoidance. However, while some informants had deep-rooted insecurities or concerns that had led to a negative personal relationship with sex, others felt that they had a positive or unhindered sexuality: “*My sexuality was (…), I didn't think there was anything weird about it. It was something exciting and something good and something positive” (IN17)*. The time around puberty, after awareness and interest in sex has started but before having sex for the first time, was described as particularly important and sensitive. Some informants described that their interest in pursuing sex or relationships was dampened by concern about both sexual function and whether they would be accepted by a partner: “*You were much, much more insecure about the first encounter with another person, of course, everyone is insecure, but I guess you were extra, maybe, because you know that you are different” (IN04).* This could extend to the point of consciously postponing any sexual intimacy. While these insecurities and concerns often decreased over time, some informants described continued patterns of avoidance.

#### 3.3.2 Having sex

Hypospadias could impact the mental aspects of having sex, the physical aspects, both, or neither. Some informants had had very good and fulfilling sex lives without any issues beyond what they viewed as “*average*”: “*There haven't been any problems (…), I've had a good sex life. It has worked great actually. You didn't think that, you know, before puberty. Or when puberty came and like (…) worrying that it wouldn't work” (IN01)*.

Examples given of physical issues included problems with ejaculation, erection, and curvature, as well as physical pain: “*Because everything was so tight, it wouldn't release the ejaculate, so it was painful, because you built up pressure that wasn't released. (…) It was unpleasant mentally and frustrating and so on. I wasn't functioning, I was having pain when others have pleasure*” *(IN15)*. Being able to sexually please a partner was viewed as an important measure of function, which some informants felt was ultimately a priority over appearance: “*I am not that big and beautiful, but I can perform so I am happy with that” (IN17)*. Some argued that while size is a touchy subject, it is ultimately not as important functionally as it may be generally perceived in society. Issues with your own sensitivity and orgasm were also highlighted, both with a partner and when masturbating. While informants pointed out that it is hard to objectively measure or compare sensitivity, the perception for some was that hypospadias surgery had decreased sensitivity to stimulation, while the other physical issues mentioned could also decrease sexual pleasure.

Function was not static over time, changing with your body but also your partner and your own mental health: “*And then we had a relationship where it's been (…) much, much, much better than it had been in a very long time. And that makes hypospadias have less and less of an impact on how I see myself as a man, in relation to a woman. That it can work like that. Physically” (IN12).* Some informants also learnt over time how certain positions or specific sexual activities helped them to for instance compensate for curvature or to achieve orgasm.

Insecurities during sex could create fear of getting reactions from partners, sometimes handled by trying to adapt how you have sex by for instance avoiding oral sex or covering up: “*[One strategy when I'm insecure] is to like use a condom. (…) It hides well, and yeah, creates like a barrier over so that it's like a win-win. You protect yourself and hide things you are embarrassed of, maybe” (IN04).* Despite the fear of partners reacting negatively, they rarely did. Some attributed it to having been with “*kind women*”. Positive experiences with sexual partners were described as healing. Those who did not identify as heterosexual did not feel that their sexuality had particularly impacted their experience in relation to hypospadias, or that the sex or gender of their partner made any clear difference.

Thoughts of when, or whether, to tell a sexual partner about hypospadias varied greatly. Some wanted to tell them straight away, some waited to see if they received a question or comment, or until they felt secure, while others did not really talk about it at all. Explanations were generally kept short, for instance “*I had surgery as a child*”, and received in a neutral or supportive way.

#### 3.3.3 Being in a relationship

In long-term relationships, hypospadias was generally described as irrelevant or unproblematic, and communication was more open. Some informants talked about experiencing sexual issues with a long-term partner, as function, sexual habits, and interest changed over time, relating to both any potential surgeries or complications, and to their lives, such as having children. Those experiences could cause great sorrow and frustration, but it could also be comforting to go through it with a partner. Finding someone secure and safe was viewed as important. For some, it was so important that being in a more secure romantic or social relationship with someone you trust was vital for sexual intimacy. Not everyone viewed being in a relationship as something they thought they wanted or could have, and some of those in a long-term relationship questioned whether they would be able to try to enter something new if they were ever single again: “*My current domestic partner and I (…), we have been together a while. So, the only thing I have thought about since then, is that if- if we were to separate at some point, would I really dare to start a new sexual relationship at some point in my life? I am very unsure whether I would” (IN11)*. Others did not consider hypospadias when starting a new relationship.

### 3.4 Public spaces

This category relates to more public spaces, including a sense of being hidden, as well as experiences relating to hypospadias concerning being naked and peeing.

#### 3.4.1 Being hidden

Informants talked about hypospadias being a hidden malformation, only visible when they are naked. Some felt that this increased their sense of shame and wished that the malformation had been anywhere else, whereas others viewed it as a positive, making it less relevant to daily and social life: “*You have your underwear on and then it's not like something you think about like if you have something- some big scar on your face” (IN12)*.

The intimate nature of hypospadias also meant that it is not something that is generally talked about, or even considered taboo. This could give a sense of isolation from your surroundings: “*You feel alone with it, as well (…). Just because it's not talked about. If you have another type of malformation, (…) maybe that would have made it easier” (IN14)*. Some actively tried to keep it secret and never really learnt how to talk about it with anyone. Others were more open to talk about it in general, could talk about it with certain people, or learnt to be more open with time.

#### 3.4.2 Being naked

Although social life in clothed spaces was generally quite unaffected, the experience of being naked in more public settings varied from unproblematic to extremely uncomfortable: “*I have been a like cool person, and then you have like your life outside of the naked world which has been really secure and then there are rooms you walk into and feel insecure. And (…) that has been tough*” *(IN04)*.

While some informants showered, bathed, and changed clothes unhindered, others talked about hiding their bodies or avoiding public nudity. However, it was pointed out that there are others who choose not to shower or to cover up as well, for instance because of shyness, or religious or cultural beliefs. For some informants, not wanting to use communal changing rooms meant avoiding sports altogether. This was particularly a problem in school, when there were less options for privacy: “*In high school, I didn't go to gym class even once, I just cut class. I have avoided sports most of my life, pretty much. I have. Now I have started to work out a bit more recently but- because then, if you go running, then you can run like at home. Then it's not the same thing” (IN14)*. Being hindered by insecurity could also have social consequences, being aware that you would not participate if someone for instance suggested swimming naked. Some informants continued to avoid situations as adults, while others grew more comfortable with age and experience.

In a public space, it became important whether any physical differences were easily visible, and how the penis looked flaccid. Setting also mattered, with some informants discussing the difference between going to a school where it is the norm to be circumcised, as compared to a more traditionally Swedish setting where circumcision is rare. Size could be particularly sensitive and the main reason for their avoidance, whereas others were more insecure about looking generally “*different*”. Some informants described actively looking at other's penises in order to try to compare their own, while comparing how you look naked was also described as something boys, or perhaps people in general, do at a certain age: “*I thought a lot about how most guys looked, and I think that has something to do with wanting to feel like it wasn't just me- that I wasn't that different” (IN11)*. Comparing could be an important normalising process, seeing that penises all look different, look like yours in some ways, or are generally unimpressive. However, it could also be a negative experience which highlighted internal perceptions of differing from the norm.

Some informants had never really expected, nor received, any reactions or comments, while others were apprehensive. Even for those who feared reactions, the issue was more in their minds and being teased was less common than they had expected. Informants could attribute this to “*kind friends*”, in parallel to “*kind women*”. For those who received comments, it was often hard to know how to react to them, and they could increase feelings of insecurity, depending on the way it was done, i.e., more mocking or hurtful comments, as opposed to neutral ones.

#### 3.4.3 Peeing

Informants described issues, especially in childhood, with peeing in a way that was different and more difficult to control, and the practical or social consequences that this could have in more public settings. Having urine flow that was at an angle or sprayed outwards made it hard to pee standing up. Even if the flow became straighter over time, there was a fear of urine ending up somewhere it should not be, making sitting and peeing more comfortable: “*Yeah, but I have probably found that a bit tough, because then I don't really know, I've been able to handle it by sitting down. (…) But it was maybe something I could think was hard when I was a kid, that- that I didn't really know how [the pee] would like come out” (IN02)*.

While some informants felt that having to sit and pee was unproblematic, others talked about the impracticality of not using urinals, and that it is more difficult to pee during outdoor activities such as camping. Some informants spoke specifically about not wanting to pee around other boys or men, for fear of them seeing that they pee differently: “*But I don't have a straight stream (…) and I didn't want to pee in a group with guys because I probably sprayed more than they did, and I probably thought that was hard, but nothing that has bothered me really” (IN05)*. In parallel to avoiding naked situations, this could make some informants feel limited in how they partook in social or physical activities. More pronounced issues peeing due to surgical complications could cause larger psychosocial issues, impacting daily life, social activities, and even work: “*Socially, it became a bit tough like having to plan to run to the toilet and like (…). It was psychologically taxing, very much so, to have to plan your entire life [around that]” (IN14)*.

### 3.5 Latent theme: varying impact and coping

We identified the latent theme *varying impact and coping*. Hypospadias presents a physical, psychological, and social challenge which all informants had to relate to in some way. As aesthetic and functional outcomes, psychology, and social contexts all differ between individuals, there is variation in the extent of the challenges faced, as well as how those challenges are met, i.e., coping. Together, differences in impact and coping form the broad spectrum of how hypospadias is experienced.

## 4 Discussion

In order to further understand the personal experience of men born with hypospadias, we conducted in-depth interviews with adult informants with a range of ages, phenotypes, and sociodemographic factors. We found that living with hypospadias was experienced both internally, in relation to identity, as well as in relation to familial, intimate, and public spaces. Within the experiences our informants portrayed, there was a large variation in the overall importance and role of hypospadias in their lives. No one described being born with hypospadias as overall positive, but it could be neutral, with hypospadias having no significant bearing on their well-being or the way they lived. However, others described hypospadias as being at the heart of recurring themes of avoidance, insecurity, and otherness in different aspects of their lives.

Other studies on health conditions in childhood and adolescence have highlighted the importance of feeling or being normal for well-being (22). Our informants described a process of realising they were different when growing out of the comparatively unaffected early stages of childhood, which was also found in a study on youths with Hirschsprung or anorectal malformations by Nah et al. (23). This aligns with the suggestion made by one of our informants that early support in normalisation could help reduce the later impacts of feeling different. Some of our informants also hypothesized that they would have struggled more or less with their confidence if they had had an issue that was visible. A study comparing adolescents with visible cleft lip and/or palate to adolescents with non-visible cleft or no cleft found that those with visible cleft reported higher levels of close friendship and social acceptance, and less depressive symptoms, than both control groups (24). The authors discussed both the psychological aspects of building emotional resilience and the effects of positive social reinforcement, which some of our informants had experienced, as possible explanations. Having a malformation that is not talked about or seen may leave fewer automatic opportunities for external moderation of internal body-image concerns.

Although issues and insecurities relating to genitalia are common regardless, hypospadias can add a level of complexity. While a Norwegian study found generally high penile perception scores (PPS) in a healthy adolescent population, men with hypospadias in a Swedish follow-up study had significantly lower average PPS than controls, decreasing further in proximal hypospadias (3, 25). Optimising surgical outcomes, with broad consideration for function and aesthetics, is one key aspect in addressing this. In relation to feeling different, it is important to consider what frame of reference boys are given by their surroundings, for hypospadias and in general. Feelings of isolation and exaggerated or incorrect conclusions could be combated by information, meaning that healthcare can help support the normalising process in boys with hypospadias and their families, to help reduce stigma, uncertainty, and shame.

While some informants had very open and supportive communication with their parents, others had more closed off or no communication, and could attribute this either to themselves, their parents or both. A recent survey study from Canadian researchers found that while 93% of parents planned to inform their children that they had undergone surgery for hypospadias, 91% had not received any guidance from healthcare professionals and 43% wished for more support in this issue (26). When comparing those with sons with distal and proximal hypospadias, proximal hypospadias was significantly more associated with being nervous about disclosure and wanting help. Supporting parents more in how to communicate about hypospadias, both with their children and in general, may further help the individual with hypospadias themselves to develop a more comfortable relationship with their condition. The key role of the mother specifically has been shown in the qualitative study by Nah on youths with Hirschsprung or anorectal malformations in the setting of East Asia (23). In our setting, it was discussed that while the father should be more directly related or better informed as hypospadias is a “male” condition, the mother still had the key role. This knowledge may be important, both in recognising the motherś function, but also that particular focus on support and involvement of fathers in healthcare may help them be more open and active.

While our informants were at different stages in their lives and had different general feelings and wishes with regards to having their own children, some informants had drawn their own conclusion that there was no link between hypospadias and fertility. A few studies have looked at fertility or semen quality, indicating that proximal hypospadias is associated with some degree of impaired fertility while it is less clear whether there is any clinically significant impact for those with distal hypospadias (12, 27–29). Informing adolescents and men with hypospadias about these findings requires a balance that allows men experiencing issues to get help and support quicker, but without raising unnecessary concern. Similarly, with regards to heritability, while there is a recurrence risk in children of fathers with hypospadias, most will have children without (4). Access to correct information may help reduce concern and give more control to the individuals, especially if there is a known genetic diagnosis or inheritance pattern which allows for more specific counselling. Some were also worried long after their child's birth that issues would arise, which could be addressed with a better general understanding of hypospadias.

Several insights were given by this study into potential impacts on intimacy and relationships. Our informants described several patterns of avoidance in approaching sex, intimacy, and relationships. Avoidant attachment styles have been reported before in men with hypospadias, especially proximal hypospadias, who in a survey study also reported being less likely to seek social support (16, 30). Apprehension in adolescence surrounding the first sexual experiences were highlighted particularly by our informants, with some feeling that they actively postponed sexual intimacy. Reassuringly, average age at sexarche did not differ between patients and controls in a Swedish follow-up study (16). Recent quantitative studies have also shown that men with hypospadias overall do not significantly differ from their peers in regard to number of sexual partners, sexual activity, or interest in sex, with more mixed results for overall satisfaction in sexual life (16, 31). The variation in our findings showed that hypospadias does not have to be a hinderance to having a satisfying sexual life, and that partners were generally understanding. The results of these quantitative studies, along with the narratives from our study, may help provide comfort and perspective for boys and men with concerns about their future. In particular, they can help address the concerns in adolescence discussed above, when some informants worried that they would never have positive sexual experiences.

For those who did experience issues with sexual function, descriptions included decreased sensitivity, difficulty with erections or orgasm, and problems with ejaculation, which is in line with previous literature (32). Pain was raised by our informants in relation to sex, both physical and psychological. Associating sexual intimacy with insecurities and discomfort or pain is a significant issue, and some informants described adapting how they have sex to try to compensate or increase pleasure. These results further show the importance of considering sexual and psychological aspects in hypospadias surgery and follow-up, as discussed in our previous article (19).

Social aspects such as avoidance of public toilets or showers are not included in any existing scales for patient reported outcomes in hypospadias (33). As raised by our informants, patterns of avoidance relating to public nudity could have large consequences, especially in childhood and adolescence, in relation to physical exercise and social interactions. Reducing these consequences is paramount. Providing normalising support to individuals and their parents could potentially help prevent avoidance. In addition, increased flexibility, autonomy, and privacy, especially in schools, may benefit some individuals with hypospadias. Our results also highlighted how extensive the social impact of severe voiding dysfunction can be. While significant issues relating to voiding are relatively rare in hypospadias, it is important to consider the social and psychological consequences for those patients, regardless of age. Insecurities surrounding the urinary stream were also discussed. During clinical follow-up, asking patients how they pee may help raise any concerns that they could benefit from discussing.

Fear of disclosure, raised by some of our informants, has been previously highlighted in studies on children and adolescents with chronic illnesses (34). For those who suffer from this fear, or wish to be more open, lack of words or guidance in how to talk about hypospadias and related matters can be a hinderance. Here as well, healthcare, patient forums, and other potential resources could provide support for those wishing to have a more open discourse with those around them. As discussed above, this openness may have beneficial effects on well-being through social support.

### 4.1 Understanding varying impact and coping

While there were similarities in the ways in which hypospadias impacted life, there was large variation in the degree of impact and coping, as described by our latent theme. Physical outcomes are clearly important, particularly in the case of poor outcomes in relation to aesthetics and urinary or sexual function. Previous research has repeatedly shown a higher degree of negative outcomes following proximal hypospadias. However, in the analysis, it was clear that individuals could experience issues relating to identity, masculinity, and forming relationships despite having a milder phenotype or relatively favourable surgical outcomes. Further, parents, partners, and friends were described by our informants as varyingly directly protective or destructive. When viewing our results as a whole, it can be interpreted that the degree of individual struggles and coping in boys and men with hypospadias relates to both individual factors such as personality, experience, and knowledge, and to interpersonal contexts. This is in line with previous research and theory on coping in children and adolescents with chronic conditions, which depends on a complex interaction between personal and interpersonal factors (35).

**Figure 3:**
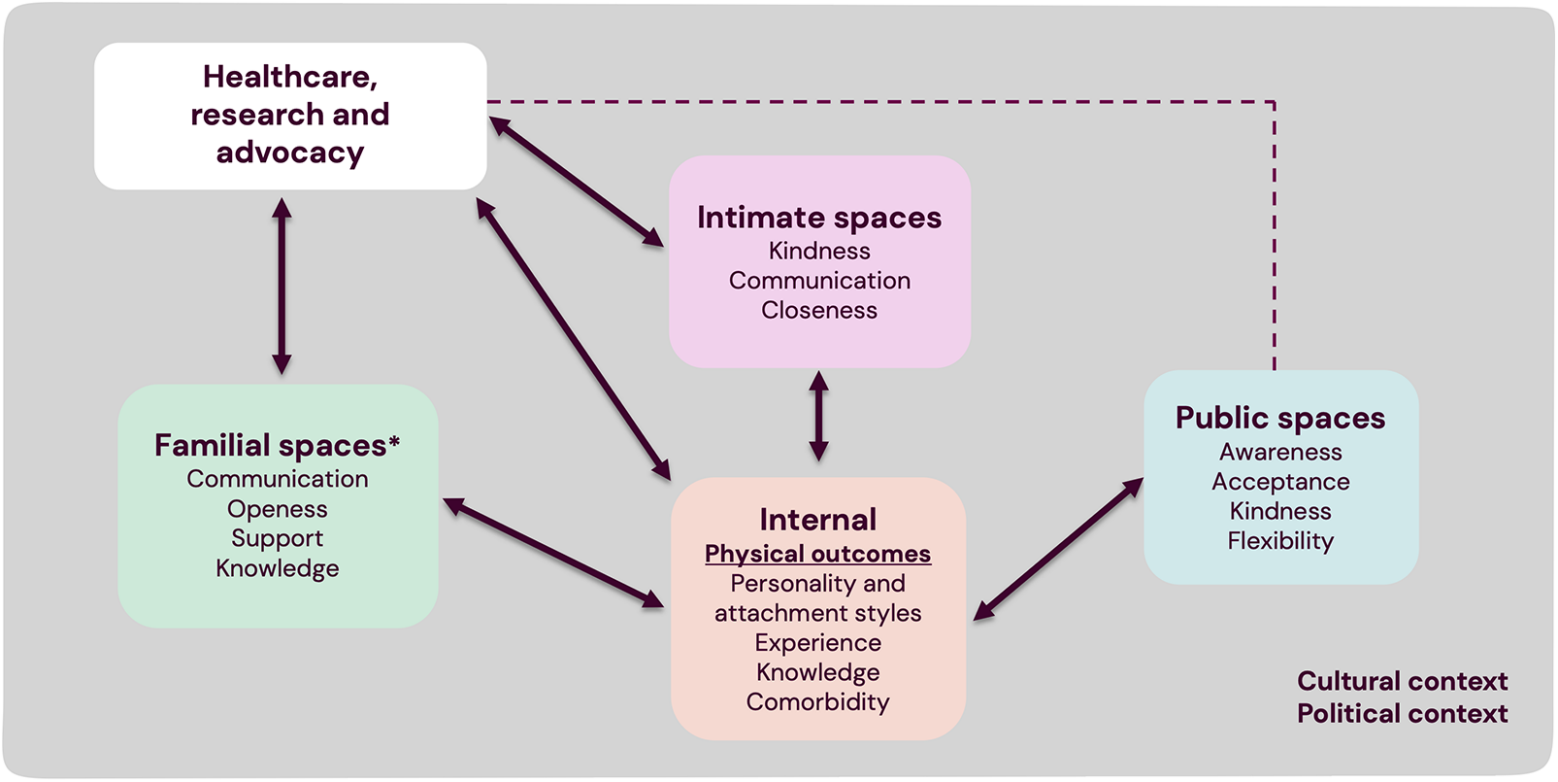
Conceptual visualisation of impact and coping in individuals with hypospadias. Physical outcomes, i.e., functional, and aesthetic outcomes of hypospadias, are highlighted due to their central importance. Other examples of factors that can relate to coping and the overall experienced impact of hypospadias are listed under internal, familial, intimate, and public respectively. These are not meant to be an exhaustive list, nor will all of these factors be important to everyone. All of these spaces may also relate to the underlying cultural and political context. Healthcare, research, and advocacy are included to show how they directly interact with and can support individuals, families, or couples. The dotted line to public space indicates less direct interaction, but in certain settings it may be relevant to for instance increase awareness or impact public policies. *Relates primarily to the family that the individual with hypospadias grows up with. The experience of starting your own family is discussed more in the results and discussion.

As well as striving to optimise surgical outcomes, intervention and support across different internal and external circumstances, as raised throughout this discussion section, may help to improve quality of life. We have summarised this visually to help understand how different factors interact (Figure 3). Our previous study presented suggestions for implementation in healthcare, including information, communication, and psychological and sexual support (19). This visualisation can complement those results as a help in identifying which individuals may be particularly vulnerable and in which ways. Both studies have highlighted puberty and adolescence as a key time period when support may be needed.

### 4.2 Strengths and limitations

This unique qualitative study provides new insight into social, sexual, and general well-being in boys and men with hypospadias. Using purposive sampling, we included a broad range of informants in order to observe variation in experiences. Our sample size is reasonable for our study design and reflects the extensive and rich data generated by in-depth interviews (36, 37). There will of course be unique experiences relating to hypospadias which may not be fully encompassed by the data from our 17 informants, but through our recruitment and study design we have been able to identify recurring overarching experiences which allowed us to create our categories and theme, as well describe the variation from positive to negative within them. The analysis was performed by the first author, who is bilingual, with repeated input throughout the process from the research group. The co-authors represent varied backgrounds which each contributed to different perspectives on the data and helped to increase understanding and improve confirmability. To help the reader to judge the credibility of our study, we have included direct quotes and examples from our data, in relation to our results.

While our informants had varying backgrounds, they all live within the same broader social and cultural context. The Swedish context will differ from many other parts of the world, which likely impacts certain experiences. The world is also constantly changing, not least in relation to the internet and social media, meaning that even within our context, some experiences may be more outdated, and more specifically modern aspects may have been missed. To help the reader to judge transferability, relevant descriptions are provided in the methods section and through the narratives of our informants.

## 5 Conclusion

This study is the first to our knowledge to use qualitative methods to study the personal experiences of men born with hypospadias in relation to the psychosocial and psychosexual. We found large variation in experiences relating to internal identity as well as familial, intimate, and public spaces. While informants generally partially shared certain experiences, such as feeling different or hidden, the overall impact varied from negligible to severe. Other than surgical outcomes and phenotype, coping and the overall impact of hypospadias relates to both personal and interpersonal factors such as personality, prior experiences, and social contexts. This means that as well as optimising physical outcomes, providing support for individuals with hypospadias and their parents who are struggling at critical time points may help to improve general well-being and quality of life.

## Data Availability

The datasets presented in this article are not readily available because the data generated in this study is highly sensitive and is therefore legally and ethically restricted. Requests to access the datasets should be directed to lottie.phillips@ki.se.
